# Short Peptide Self-Assembly in the Martini Coarse-Grain Force Field Family

**DOI:** 10.1021/acs.accounts.2c00810

**Published:** 2023-03-03

**Authors:** Alexander van Teijlingen, Melissa C. Smith, Tell Tuttle

**Affiliations:** Pure & Applied Chemistry, University of Strathclyde, 295 Cathedral Street, Glasgow G1 1XL, U.K.

## Abstract

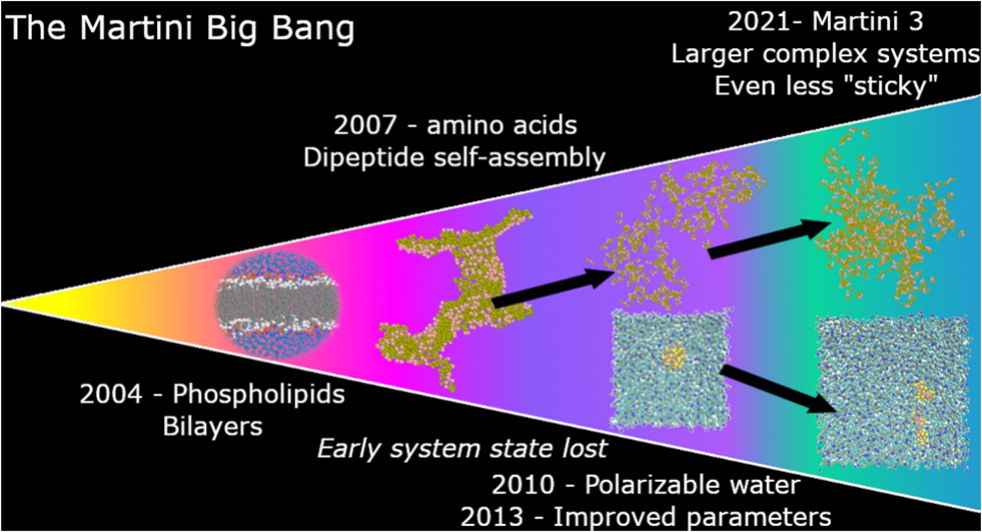

Pivotal to the success of any computational experiment is the ability to make reliable predictions about the system under study and the time required to yield these results. Biomolecular interactions is one area of research that sits in every camp of resolution vs the time required, from the quantum mechanical level to *in vivo* studies. At an approximate midpoint, there is coarse-grained molecular dynamics, for which the Martini force fields have become the most widely used, fast enough to simulate the entire membrane of a mitochondrion though lacking atom-specific precision. While many force fields have been parametrized to account for a specific system under study, the Martini force field has aimed at casting a wider net with more generalized bead types that have demonstrated suitability for broad use and reuse in applications from protein–graphene oxide coassembly to polysaccharides interactions.

In this Account, the progressive (Martini versions 1 through 3) and peripheral (Sour Martini, constant pH, Martini Straight, Dry Martini, etc.) developmental trajectory of the Martini force field will be analyzed in terms of self-assembling systems with a focus on short (two to three amino acids) peptide self-assembly in aqueous environments. In particular, this will focus on the effects of the Martini solvent model and compare how changes in bead definitions and mapping have effects on different systems. Considerable effort in the development of Martini has been expended to reduce the “stickiness” of amino acids to better simulate proteins in bilayers. We have included in this Account a short study of dipeptide self-assembly in water, using all mainstream Martini force fields, to examine their ability to reproduce this behavior. The three most recently released versions of Martini and variations in their solvents are used to simulate in triplicate all 400 dipeptides of the 20 gene-encoded amino acids. The ability of the force fields to model the self-assembly of the dipeptides in aqueoues environments is determined by the measurement of the aggregation propensity, and additional descriptors are used to gain further insight into the dipeptide aggregates.

## Key References

- FrederixP. W. J. M.; UlijnR. V.; HuntN. T.; TuttleT.Virtual Screening for Dipeptide Aggregation: Toward Predictive Tools for Peptide Self-Assembly. J. Phys. Chem. Lett.2011, 2, 2380–238410.1021/jz201057323795243PMC3688361.^1^*The Martini force field was used to simulate all gene-encoded dipeptides, finding good agreement between simulated aggregation and experimental results. Furthermore, the suprastructure of FF is simulated over a longer period of time, producing a nanotube with a water pore in accordance with previous experimental investigations.*
- FrederixP. W. J. M.; ScottG. G.; Abul-HaijaY. M.; KalafatovicD.; PappasC. G.; JavidN.; HuntN. T.; UlijnR. V.; TuttleT.Exploring the sequence space for (tri-)peptide self-assembly to design and discover new hydrogels. Nat. Chem.2015, 7, 30–3710.1038/nchem.212225515887.^2^*Screening of all tripeptides derived from gene-encoded amino acids within the Martini force field for aggregation. A particular focus is given to soluble self-assembling peptides that are driven not only by hydrophobic interactions.*
- Van TeijlingenA.; TuttleT.Beyond Tripeptides Two-Step Active Machine Learning for Very Large Data sets. J. Chem. Theory Comput.2021, 17, 3221–323210.1021/acs.jctc.1c0015933904712PMC8278388.^3^*Combining active machine learning and the Martini force field to extend virtual screening to much larger data sets (up to hexapeptides). The generation of machine learning descriptors enables data set filtration to direct the machine learning toward particular targets (e.g., solubility).*
- Van TeijlingenA.; SwansonH. W. A.; LauK. H. A.; TuttleT.Constant pH Coarse-Grained Molecular Dynamics with Stochastic Charge Neutralization. J. Phys. Chem. Lett.2022, 13, 4046–405110.1021/acs.jpclett.2c0054435486900PMC9109222.^4^*Developing a constant-charge pH algorithm and accurately reproducing the experimental pH-dependent behavior of two peptide systems.*

## 1 Introduction

Coarse-graining is an extremely useful tool in the computational chemist’s toolkit. It enables the emergence of properties of macromolecular systems that cannot be practically captured on shorter time scales. Macroscale properties often emerge over time scales infeasible for all-atom molecular dynamics (MD). However, coarse-grain (CG) simulations enable longer time scales by increasing the time step, determined by the lightest particle in the system, and reducing the number of arithmetic calculations by reducing the number of beads and degrees of freedom.^5^

The Martini force field is one of the most popular CG force fields and provides a generalized formula that avoids needing to reparameterize for each system under investigation.^6,7^ This is due to the versatility offered by the force field in the fields of biochemistry, materials science, and mesoscale modeling.^8,9^ Pezeshkian et al. demonstrated the versatility of the Martini force field with their simulation of an entire mitochondrial membrane by employing the Dry Martini force field where water is not explicitly simulated but instead phospholipids are parametrized to imply an aqueous environment.^10^ In the same year, Martini (v2.2) was used to study the SARS-Cov-2 virus. By exploiting the increased time and sizes scales inherent in CGMD, the entire viral envelope was able to be simulated.^11−14^ These examples show the scale of simulations possible, which remain inaccessible to all-atom MD.

There have been several iterations of the Martini force field aiming to improve macromolecular simulations (proteins in phospholipid bilayers, etc.) and thermodynamic properties such as free-energy transfer across a bilayer. For example, Majumder et al. demonstrated that a scaling factor based on the difference between the experimental and computationally derived free energy of dimerization of four proteins was able to decrease protein–protein aggregation within a bilayer.^15^ However, modifications that prevent membrane proteins from aggregating by decreasing the interaction strength can have negative unintended consequences whereby previously well described interactions between constituents of small systems are lost, though until now this is something yet to be thoroughly explored.^6^ Changes in particle definitions and their associated terms can affect many aspects of the simulations. Vitalini et al. has demonstrated that peptides switch between conformational wells at rates differing by up to 2 orders of magnitude depending on which force field is used to model them.^16^

In previous studies, we have found that within the Martini 2.1 force field many dipeptides did aggregate in aqueous environments.^1^ However, when we used the Martini 2.2 force field to measure the same effect we found far less aggregation taking place.^3^ This is consistent with the assertion that progressive iterations of the Martini force field have lost accuracy in short (two to three amino acids) peptide aggregation by making amino acids less “sticky” to better represent larger systems. Herein we aim to explore the consequences of the different Martini force field parameters on dipeptide aggregation in terms of Lennard-Jones (LJ) nonbonded (NB) parameters and intramolecular potentials (bonds, angles, and dihedrals).

### 1.1 Coarse Graining

The first attempt at CGMD was conducted by Levitt et al., who developed a simplified model for protein folding,^17^ with later work by Smit et al. in 1990 using CG models to simulate multiphase (water/oil) interface behavior.^18^ While these earlier examples demonstrated the potential of CG models, it was not until 2004 that Martini emerged and rapidly became one of the most popular CG force fields for modeling lipids and proteins.^6,19^ CGMD allows simulations to run faster by reducing the number of particles in the system and their associated degrees of freedom. Paired with evaluating only short-range interactions and a larger time step, the simulation speed of CGMD simulations is 2 to 3 orders of magnitude faster than atomistic simulations.^20^

The use of a relatively small number of beads to model proteins, lipids, solvents, and small organic molecules and the simplicity of the force field using standard interaction potentials^19^ have contributed to the wide adoption of the Martini force field. However, in order to dynamically represent amino acid secondary structure in this approach, additional development of the original force field was required.^21^ One approach, proposed by Matysiak et al., introduces dipoles on the backbone beads of a Martini-derived force field and has been shown to reliably predict α/β content in various proteins.^22,23^

### 1.2 Beads

The Martini force fields use a standard 4:1 mapping in heavy atoms to beads, with smaller (3:1) and tiny (2:1) beads also available. The beads range in polarity and relative attractiveness to each other in order to capture the range of nonbonded interactions in organic and biological systems, as well as the Drude polarizable water (PW) model that has its own bead types. These bead types and the Martini force fields in which they are implemented are described in Table 1.

**Table 1:** Effective Size Is Given as the Self–Self LJ σ Value^24^

	Martini 2.1/2.1P/2.2/2.2P		Martini 3	
	mass (amu)	effective size (nm)	mass (amu)	effective size (nm)
normal	72	0.47	72	0.47
small	45	0.43	54	0.41
tiny	N/A	N/A	36	0.34
PW (POL/D)	24	0.47/0.0	N/A	N/A

**Figure 1:**
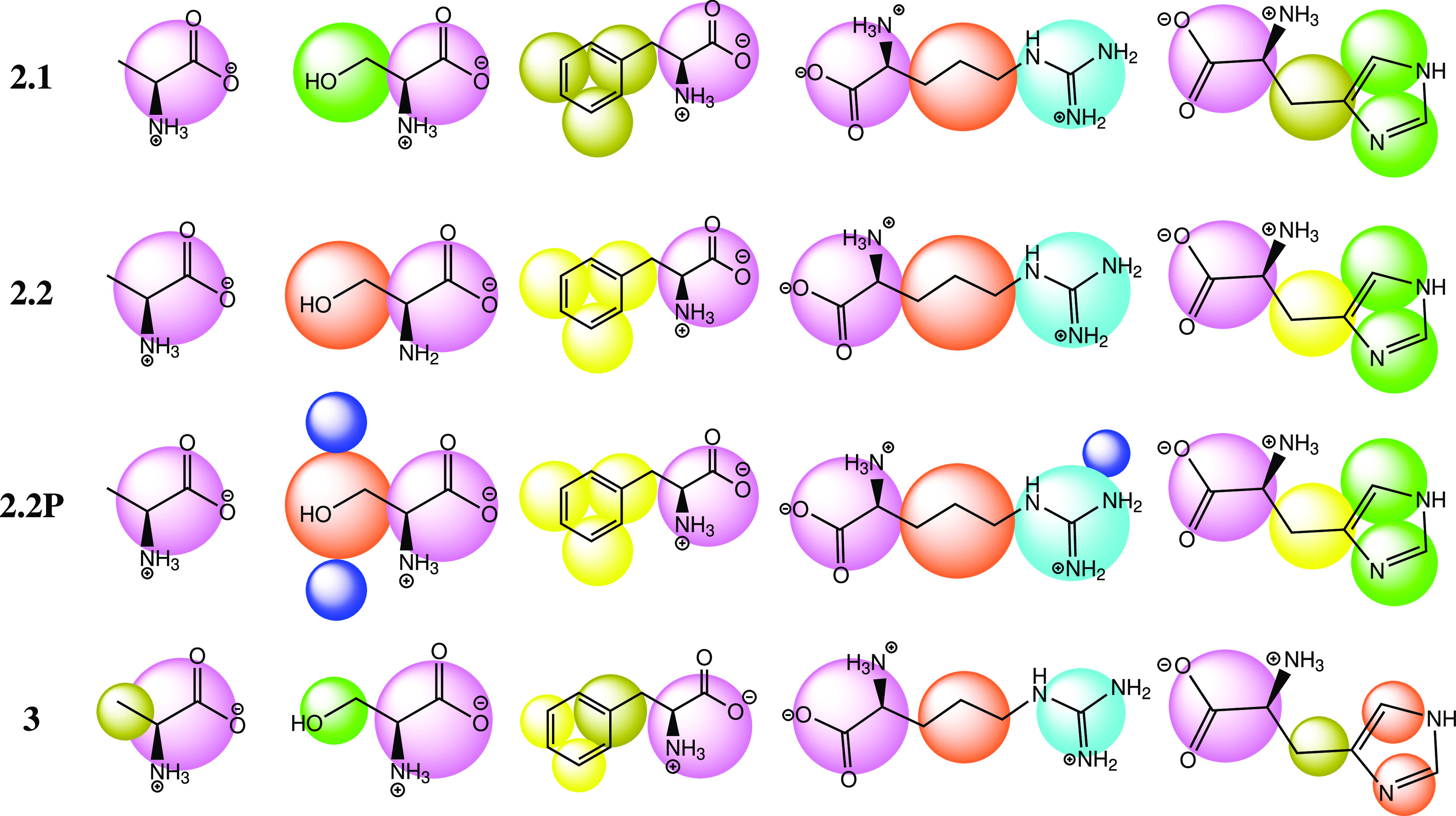
CG representations of amino acids in different versions of the Martini force fields. Dark blue, dummy beads; light blue, charged; green, polar; orange, nonpolar; dark yellow, apolar; yellow, less apolar. Sizes are relative and show the standard, small, and tiny beads.

Martini 2.1 and 2.2 have the same beads and terms, but some of the amino acids are defined differently (Figure 1). Martini 2.2P (and 2.1P) have the addition of the central polarizable water bead (POL) and the dummy bead (D) and have modified bead LJ terms for the four charged beads.^25^ Martini 3 has completely redefined amino acid definitions with new bead types and redefined bead parameters.^6^

For example, alanine has a side chain (SC) represented by a tiny bead in Martini 3 whereas in all previous Martini 2 versions (2.1, 2.1P, 2.2, and 2.2P, hereafter referred to as Martini2*) the SC is absorbed into the backbone (BB). This increases the number of beads and is a step closer to all-atom accuracy, but the time step must be decreased to accommodate smaller beads. Also, with more beads there are more terms to evaluate at each step. The default BB beads for dipeptides in Martini 2* are a charged donor and acceptor (N-terminus and C-terminus, respectively), which have an attractive preference toward each other, in line with intuition. However, Martini 3 changed the default to two charged beads, which have no preference for N-terminus to C-terminus alignment over other mutual charge arrangements.

In all Martini models, normal water beads represent four water molecules following the same mapping ratio for computational efficiency. Martini 3 includes “small” (SW)- and “tiny” (TW)-sized water, the effect of which will also be investigated in terms of the self-assembly nanodisc in an aqueous environment. In the Martini 2.1P/2.2P model, water molecules are represented by a core bead attached to two oppositely charged dummy beads (which sum to a mass in the same way as the regular water bead). These polarizable waters and amino acid side chains are based on the Drude model.^25^ Antifreeze water molecules are also available to prevent freezing in ordered surfaces such as phospholipid bilayers^20^ or surfaces such as graphene.^26^ Antifreeze water does not affect dipeptide self-assembly, so it has not been studied here. Martini versions that aim to speed up simulations but not change the simulation outcome are discussed in the Supporting Information, section 1.

### 1.3 Bonds and Angles

Dipeptides have the same bonds, angles, and constraints within the Martini 2* family of force fields but have been changed in Martini 3. These changes, while seemingly small, are important for the reproduction of other physical properties and affect the self-assembly behavior of short peptides (two to three amino acids). In particular, how the tightening of the angle between the BB and aromatic SCs and the angles within aromatic SCs, the lengthening of the bonds between the BB and SCs, and how the increase in the force constant between BB beads affects dipeptide self-assembly are explored.

An alternative approach to using harmonic bonds was developed by Poma et al., replacing bonded terms with LJ potentials. This approach, called G o̅Martini, allows for sampling unfolded and folded protein states dynamically within the Martini force field and capturing the motion key to catalytic activity, in good agreement with all-atom simulations.^27^

## 2 Self-Assembly within the Martini Force Field

Our laboratory’s first venture into investigating self-assembly with Martini was in 2011 with the virtual screening of all 20^2^ gene-encoded dipeptides in their zwitterionic state using Martini 2.1. This laid the groundwork for rapid nonspecific self-assembly of short peptide sequences using CGMD with the Martini force field. This method was validated at the time by alignment with experimental results that had shown different dipeptides’ ability to aggregate in aqueous environments.^1^

Four years later, the entirety of the tripeptides (20^3^) sequence space was simulated in their zwitterionic state using Martini 2.1. At this sequence length, zwitterionic peptides with a net charge are able to aggregate in water, which was not observed in dipeptides. This study also introduced a descriptor for scoring aggregation while also accounting for the solubility of the peptide through their log *P*.^2^ This helped to distinguish between those peptides that would aggregate and potentially precipitate out of solution and those that would be able to potentially self-assemble and remain in solution to form a nanofibrous network capable of supporting a hydrogel.

Some of these peptides have been further investigated for other interesting properties and novel self-assembly patterns. Two groups of amphiphilic tripeptides—KFF, KYF, and KYW as well as DFF and FFD—were found to be effective emulsifiers, though operating through two distinct methods. The cationic tripeptides formed fibrous networks around an oil droplet in water, and the anionic tripeptides acted as a surfactant.^28^

When the search space was explored further, a study of coassembling tripeptides was conducted on the properties of GHK and FFD nanostructures. While FFD formed bilayer-like aggregates and GHK formed random aggregates, together they self-assembled into tape-like structures that trapped water within its structure.^29^ It was discovered experimentally that upon the addition of CuCl_2_ the tape-like structures rapidly nucleated to form spherulite-like networks of nanofibers, thus demonstrating how ions affect the cooperative assembly of peptides in solution.

In 2016, Guo et al. performed a virtual screening which focused on the coassembly properties of FF/FFF systems by the relative concentration to produce a wide array of macrostructures from toroids to nanovesicles to collapsed spheres using the Martini 2.1 force field.^30^ This provided an inspiration for our laboratory to measure the effect of introducing the much-studied DFF tripeptide to each of the dipeptide systems. This study highlights how cooperative assembly can yield structures of greater order than the sum of its parts; e.g., DFF forms nanodisc structures and SW forms random aggregates while DFF + SW forms nanofibers with an AP greater than either of the structures produced by its components (AP: DFF = 2.25, SW = 1.7, and DFF + SW = 2.4). Conversely, it was also shown that the addition of an unfavorable dipeptide (EK) could prohibit the self-assembly of DFF (AP: EK = 1.0 and DFF + EK = 1.3).^31^

In 2021, we increased the magnitude of peptide virtual screening by several orders of magnitude to the hexapeptide range (20^6^) using an active machine learning algorithm and Martini 2.2. The algorithm can reduce the search space to abide by user-defined constraints such as only searching for the best aggregators that are also water-soluble (log *P* < 0) and thus are not simply precipitating. This method was successful in predicting nonintuitive candidates such as WGGGGC and YYKDC as potential self-assemblers.^3^

In 2020 “Sour Martini” was developed by Grünewald et al. which emulates proton exchange within the Martini 3 force field. Capable of simulating from pH 3 to 8 via charged dummy beads, this model was able to reproduce the increase in apparent p*K*_a_ of oleic acid micelles as well as the radius-protonation relationship within the dendrimer poly(propyleneimine), demonstrating the radial shrinking as a function of pH.^32^

In 2022, we extended the landscape of our self-assembly predictions by developing a method of performing constant-charge, constant-pH CGMD (CpHMD) based on the method of Radak et al.^33^ Given that several previous experimental studies had indicated that the charge state of small molecules was changed upon aggregation, we reasoned that, to properly capture the driving forces behind self-assembly, this adjustment in charge needed to be captured by the model. This method was used to reproduce the experimental results of Adams et al., whereby 9-fluorenylmethoxycarbonyl-Phe-Phe (FmocFF) nanotubes are stable at neutral and basic pH but undergo syneresis under acidic conditions.^34^ Our model was also able to reproduce the two shifted apparent *pK*_a_ values of the C-termini of FmocFF reported by Tang et al.^4,35^

The use of CGMD to search for properties related to self-assembly has also been demonstrated via screening for tetrapeptide emulsifiers that do not contain aromatic amino acids. Using Martini 2.1, zwitterionic peptides were equilibrated in water/octanol for 100 ns. By measuring the % adsorbance (%ADS) of a peptide sequence at the interface, it was determined that alanine and arginine residues contributed the most and least to %ADS, respectively. Based on this initial screening, a series of peptides were simulated for 10 μs and produced a series of peptides that were then investigated experimentally. It was concluded that this screening method could correctly discriminate between nonaromatic tetrapeptides with high and low surface activity.^36^

In 2022, our group produced a Martini 2.2-compatible coarse-grained graphene oxide (CGGO) model. This was used to study the coassembly of proteins and stacks of CGGO. By using Martini 2.2 instead of 2.1, we could delineate between protein aggregation (not observed) and protein–CGGO aggregation (observed). This model correctly predicted the confining effect of the proteins on the CGGO interlayer distance and revealed why a 70% ethanol solution produced the most reduced graphene oxide upon heat treatment, being that this solution produced the conformational changes in the protein that squeezed the interlayer distance the most. This finding was confirmed by subsequent WAXS experiments.^26^

## 3 Dipeptide Self-Assembly Ministudy

### 3.1 Diphenylalanine

Researchers have studied diphenylalanine (FF) in many settings,^37−43^ largely due to its presence in larger biological systems and its role in self-assembly behavior,^44,45^ especially within the amyloid^46^ protein and other proteins responsible for neurodegenerative diseases.^47^ This has led to its development in applications as broad as piezoelectronics to carbon nanotube bioconjugates.^42,48^ As mentioned, self- and cross-terms between beads within the Martini 2.1 and 2.2 force fields are the same, but the choice of beads to represent some amino acids, notably phenylalanine, differs. The change was prompted by the work of Singh et al., who demonstrated that in comparison to the experimental data^49^ the Martini 2.1 representation of phenylalanine produces a divergence in the partitioning free energy of the POPC/water interface.^50^ This contributed to Martini 2.2 having redefined the representation of the phenylalanine SC. The representation of phenylalanine was further modified in Martini 3 (Figure 1).

These changes, however, had negative consequences for the self-assembly of FF in aqueous media. For example, Gazit et al. demonstrated experimentally how a slight change in phenylalanine (phenylglycine) produced markedly different self-assembly behavior (tubular → spherical).^38^ Likewise, we see how small changes in bead definition, angles, and bond lengths can yield strikingly different simulation outcomes.

**Figure 2:**
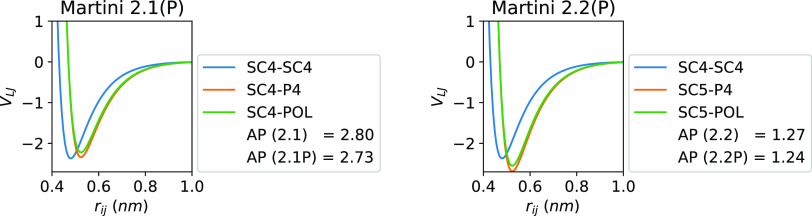
LJ interaction terms of F SCs in the Martini 2* force fields. For the polarizable water models, only the POL bead is considered as the D beads are LJ-invisible.

We find that in the case of the redefinition from Martini 2.1 to 2.2, while keeping all other variables the same and using the standard Martini water model, the AP drops to the non-assembler range (Figure 2). While the SC-SC self-interaction between these models is the same, the SC bead of phenylalanine in Martini 2.2 is slightly more polar and interacts more strongly with water. Given that Martini 2.2 benzene forms a separate nonaqueous phase, we can deduce that the backbone provides sufficient hydrophilicity to see that this change in SC beads is enough to negate the self-assembly observed experimentally.

**Figure 3:**
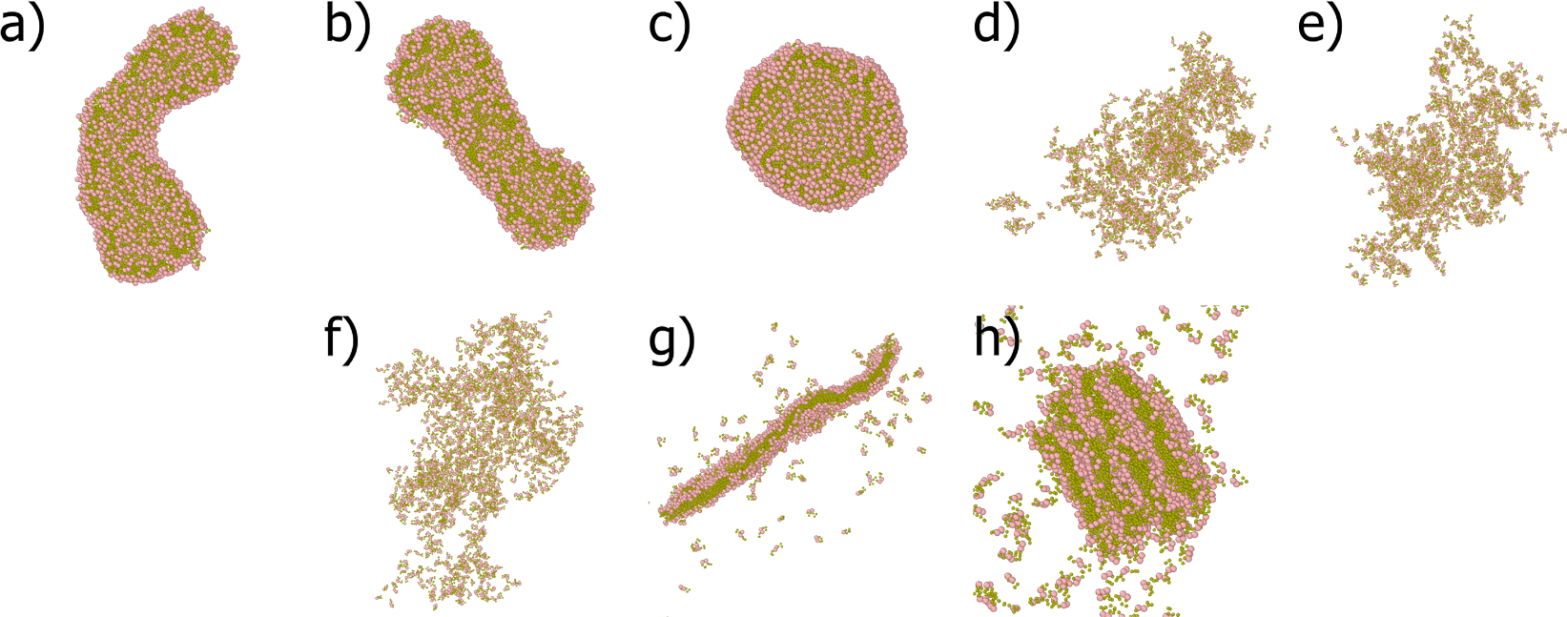
1200 FF simulations at 4 μs, in which pink beads represent peptide backbones and dark-yellow beads represent the side chains, for Martini versions (a) 2.1, hollow capped nanotube, (b) 2.1^*b*^, hollow capped nanotube, (c) 2.1P, increase in BB·BB interactions resulting in a pinker surface and a spherical membrane bilayer, (d) 2.2, no aggregation, (e) 2.2P, no aggregation, (f) 3, no aggregation, (g) 3SW, formation of a nanodisc, and (h) 3TW, formation of stacked nanodiscs.

The polarizable version of the Martini 2* force fields does not change any phenylalanine SC bead, thus the addition of polarizable water has a negligible effect on the AP score. However, it does alter the morphology of the self-assembled structure of FF from tubular to spherical (Figure 3a,c) by increasing the degree of hydrogen bonding between residues (Table 2). To demonstrate these force field effects, we simulate all dipeptides as zwitterions (300 in a 12 nm^3^ box) for 200 ns in each Martini force field and FF as larger systems with 1200 peptides in a 20 nm^3^ box for 4 μs. Computational methods for each system investigated can be found in the Supporting Information, section 2

**Table 2:**
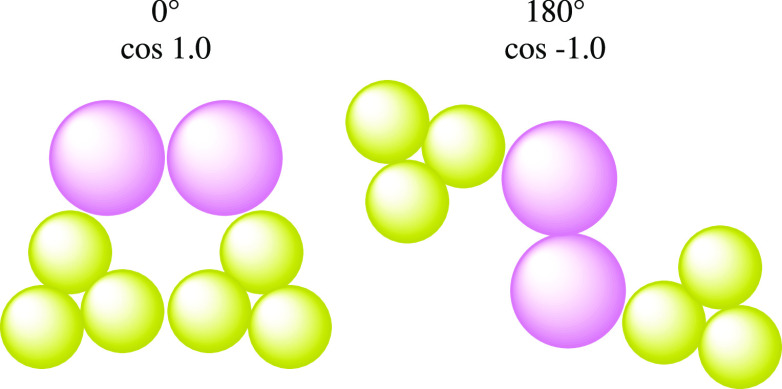
Metrics for Triplicate 200 ns Simulations of 300 FF Peptides in an Aqueous Environment, Averaged over All Molecules/Bonds^a^

	cos eq. dihedral	cos min. dihedral	BB–SC (Å)	SC–SC (Å)	AP	*R*_g_ (Å)	HB%^e^
*t* = 0 ns					1.0 ± 0.0	62.9 ± 0.9	0.0 ± 0.0
2.1	0.8 ± 0.3	–0.1 ± 0.7	3.05 ± 0.12	2.70	2.80 ± 0.09	45.4 ± 1.0	3.33 ± 0.00
2.1^b^	0.8 ± 0.3	–0.3 ± 0.5	3.20 ± 0.13	3.23	2.58 ± 0.03	48.6 ± 9.8	1.83 ± 0.83
2.1P	0.8 ± 0.2	–0.3 ± 0.6	3.02 ± 0.12	2.70	2.73 ± 0.03	60.4^d^ ± 0.4	19.61 ± 2.38
2.2	0.4 ± 0.6	–0.2 ± 0.6	3.05 ± 0.12	2.70	1.27 ± 0.03	60.9 ± 2.0	0.67 ± 0.00
2.2P	0.5 ± 0.6	–0.4 ± 0.6	3.04 ± 0.12	2.70	1.22 ± 0.01	60.4 ± 0.2	4.50 ± 1.08
3	0.2 ± 0.7	–0.4 ± 0.6	3.22 ± 0.14	3.23	1.07 ± 0.00	63.0 ± 1.2	0.00 ± 0.00
3^c^	0.8 ± 0.2	0.9 ± 0.1	3.23 ± 0.13	3.23	1.03 ± 0.01	63.1 ± 1.0	0.00 ± 0.00
3SW	0.7 ± 0.4	–0.3 ± 0.6	3.25 ± 0.14	3.23	2.57 ± 0.12	63.8 ± 0.2	0.89 ± 0.63
3TW	0.6 ± 0.5	–0.4 ± 0.6	3.24 ± 0.12	3.23	1.98 ± 0.04	63.9^d^ ± 0.1	1.11 ± 0.57

a Dihedrals are given as their cosine values with values closer to 1.0 usually indicating greater aggregation, except where they have been induced by the addition of a dihedral term (3, footnote c). SC–SC bonds are constrained; therefore, the very small deviations is not listed.

b 2.1 Martini 3 bond/constraint distances to demonstrate the bond length effect.

c Weak dihedral term applied to make the equilibrium monomer structure similar to 2.1.

d *R*_g_ decreases relatively for the 1200 monomer 4 μs simulations in Figure 3c and 3h, respectively.

e Hydrogen bonding percentage, a metric derived from that reported by van Lommel et al.^51^

**Figure 4:**
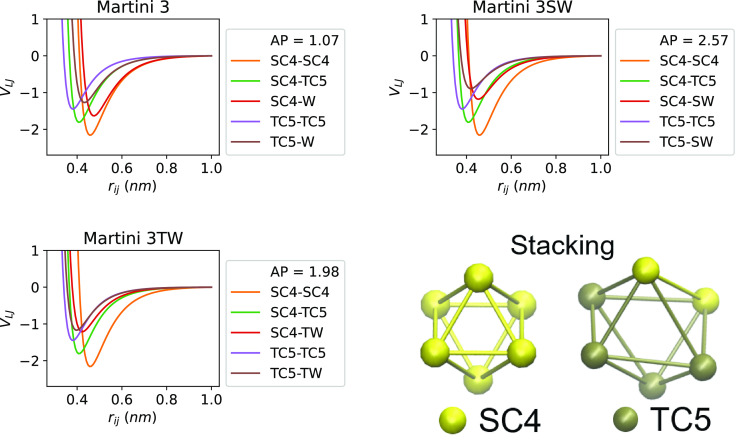
LJ interaction terms of F SCs in the Martini 3 force fields. The inset is the minimized π-stacked structure of the F SC with a comparison between the Martini 2.1 (left) and 3 (right) versions.

Comparing the CGMD simulation results of Martini 2.1 with Martini 3 is slightly more complicated due to having not only different SC beads but also different BB beads and different LJ terms for the same bead, bond, angle, constraint, and dihedral terms. Upon inspection, the LJ terms suggest that diphenylalanine would aggregate in Martini 3 water. However, the use of differently sized beads in the SC (small and tiny) prevents neat packing as the SC bond lengths are constrained such that the tiny beads (TC5) cannot reach the thermodynamic minimum (−1.45 kJ mol^–1^) without coproducing a repulsive term in the small beads (SC4, Figure 4). This SC stacking thermodynamic minimum has been visualized in the inset of Figure 4 for both the Martini 2.1 and 3 models. These specific interactions produce an overstabilized π-stacking effect in Martini 2.1, which minimizes to an LJ minimum of −21.0 kJ mol^–1^ as compared to CCSD(T) in the gas phase with values of −7.5 to −11.7 kJ mol^–1^.^52^ We find that the Martini 3 stack produces a much more accurate π-stacking energy of −12.7 kJ mol^–1^. This agreement with lower-level observations does point to the heart of the problem, in that to attain structures observed from dipeptide self-assembly within a computationally feasible time scale it may be necessary to accentuate their interactions.

In investigating the effects of the small and tiny water beads in Martini 3 on FF self-assembly, we find that the reduced SC–water interaction energy decreases the solubility of the phenylalanine residue and increases the packing of individual water beads due to the shorter self-interaction distance (Figure 4) which proportionally increases aggregation. However, the unfavorable packing of the SC beads still exists, and as such, the aggregation behavior is distinctive from that of Martini 2.1. This has been visualized in Figure 3a,g,h, where instead of a tubular structure, a nanodisc and stack of nanodiscs are formed. These nanodiscs have a broader distribution of SC-BB-BB-SC dihedral angles and a lower HB% between the BB beads (Table 2), which indicates that the characteristic π-stacking and hydrogen bonding of a diphenylalanine nanostructure are weaker. Thus, Martini 3 results in a more disordered and purely hydrophobically driven aggregation of diphenylalanine.

**Figure 5:**
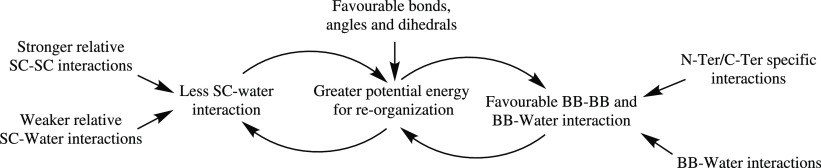
Martini 3 has relatively stronger SC–SC interactions, when using the small or tiny water models, compared to the SC–water interactions, which helps to precipitate peptides and orientated SC toward each other. Martini 2.1/2.1P have the more favorable bond and angle terms which allows reorganization toward each other for a lower energetic penalty such that the SC–water NB term is almost comparable to the SC–SC NB term. In either case, once reorientation away from water begins to occur, it lessens the interactions with water, which allows for greater reorganization.

The driving forces discussed can be summarized by Figure 5. We have attempted to describe these processes as a concise series of interactions. However, the process is dynamic, and there is a continuous interplay between the different elements of the peptide and water interactions.

Alessandri et al. observed that shorter bond lengths tend to produce more hydrophobic behavior within the Martini force field. This has been dubbed the “bond length effect”.^24^ Martini 2* assigns shorter bond lengths to phenylalanine residues, and indeed we find that changing the bond lengths to those of the Martini 3 definition while keeping the same beads and force field (2.1) slightly decreases the AP score yet retains the morphology (Table 2, 2.1^*b*^ and Figure 3a,b). In another attempt to increase the number of controlled variables, we add an explicit harmonic dihedral around 0° at 5 kJ mol^–1^ to determine if this would prompt self-assembly without having to change the peptide beads. However, it seems that unless the conditions are such that optimal dihedrals arise by themselves, it does not matter if it is present or not (Table 2, 3^*c*^).

### 3.2 Martini 2.1 vs 2.2 vs 3

**Figure 6:**
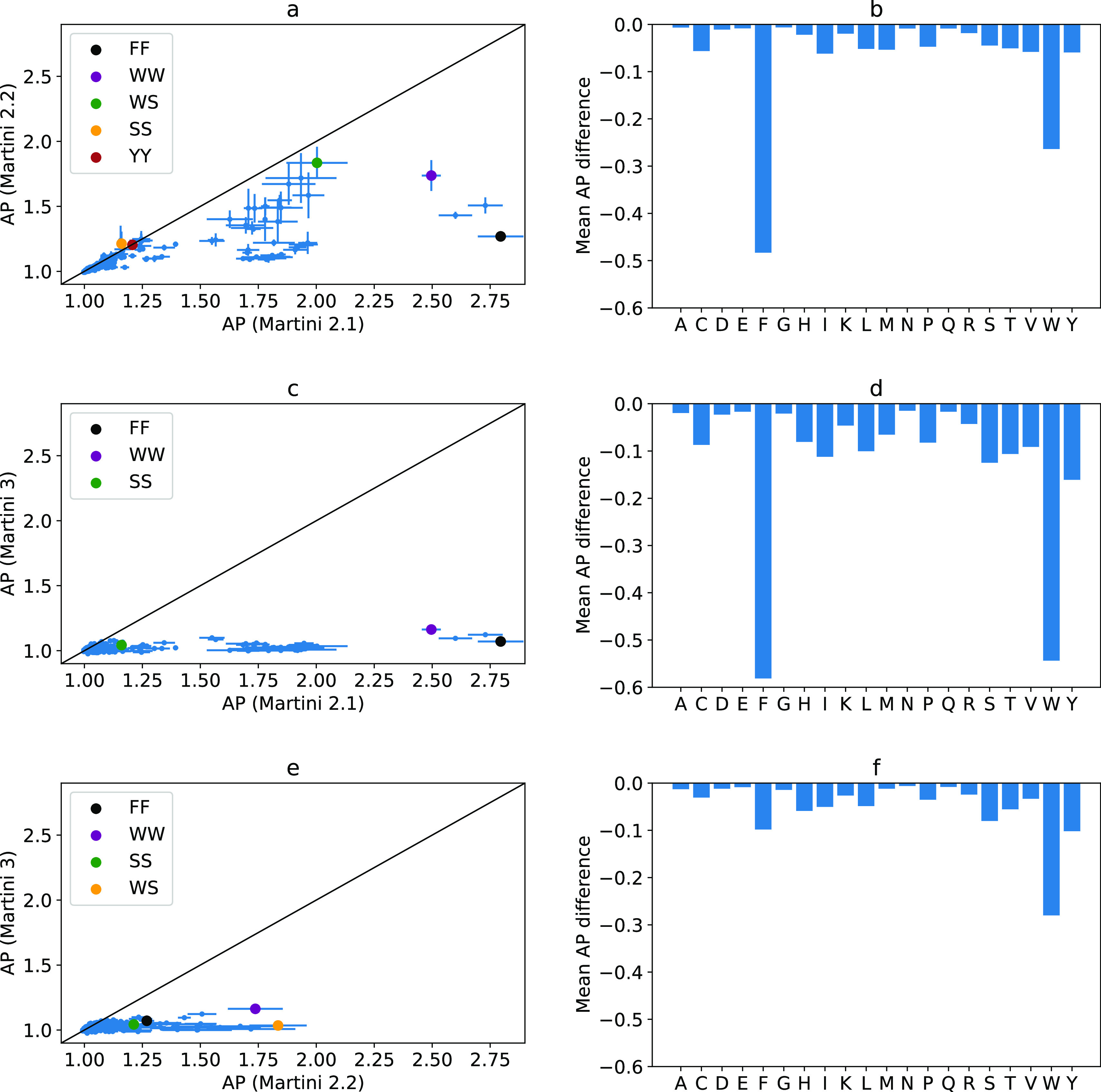
(a) Comparison between AP scores of Martini 2.1 and 2.2. (b) Difference in AP score contribution per amino acid. (c) Comparison between AP scores of Martini 2.1 and 3. (d) Difference in AP score contribution per amino acid. (e) Comparison between AP scores of Martini 2.2 and 3. (f) Difference in AP score contribution per amino acid.

In comparing broadly the three versions of Martini with standard water, we find markedly different results (Figure 6). Looking first to Martini 2.1 and 2.2, a correlation is observed for part of the dipeptide spectrum, particularly around the nonassemblers and those not containing phenylalanine or tryptophan residues (Figure 6a,b). This can be largely explained in terms of the polarity of the residues, as described for phenylalanine. The same effect is observed for tryptophan, where in Martini 2.2 the balance in the SC beads’ polarity leads to increased interaction strength with water beads. Where these two force fields produce similar AP scores is where the amino acid definition has not been changed (e.g., tyrosine) or where one residue induces a negative AP change and the other induces a positive one (e.g., WS, Figure 6a).

In comparing Martini 2.1/2.2 with Martini 3, the most direct comparison occurs when using standard water, where we observe that dipeptides do not aggregate in Martini 3 (Figure 6c–f). This is an effect of the water model which does not promote self-assembly with the increased prevalence of small and newly introduced tiny beads in the peptide models (*vida infra*).

### 3.3 Polarizable Water

It must be noted that no amino acids in Martini 2.1P contain polarizable groups, and thus the difference between 2.1 vs 2.1P is solely the effect of the PW solvent. This can help give a better understanding of the effect of polarizable water. By reducing ε_r_ from 15 to 2.5, the standard procedure when using Martini PW, the magnitude of electrostatic interactions is increased. This has the effect of increasing the water contact surface of the aggregate between the charged BB beads and the charged PW molecules, resulting in a spherical water-containing aggregate (Figure 3a,c) and an increased HB% which is in line with the findings of Piskorz et al.,^53^ who found Martini 2.2P to form more hydrogen bonds during self-assembly than any other force field tested.

**Figure 7:**
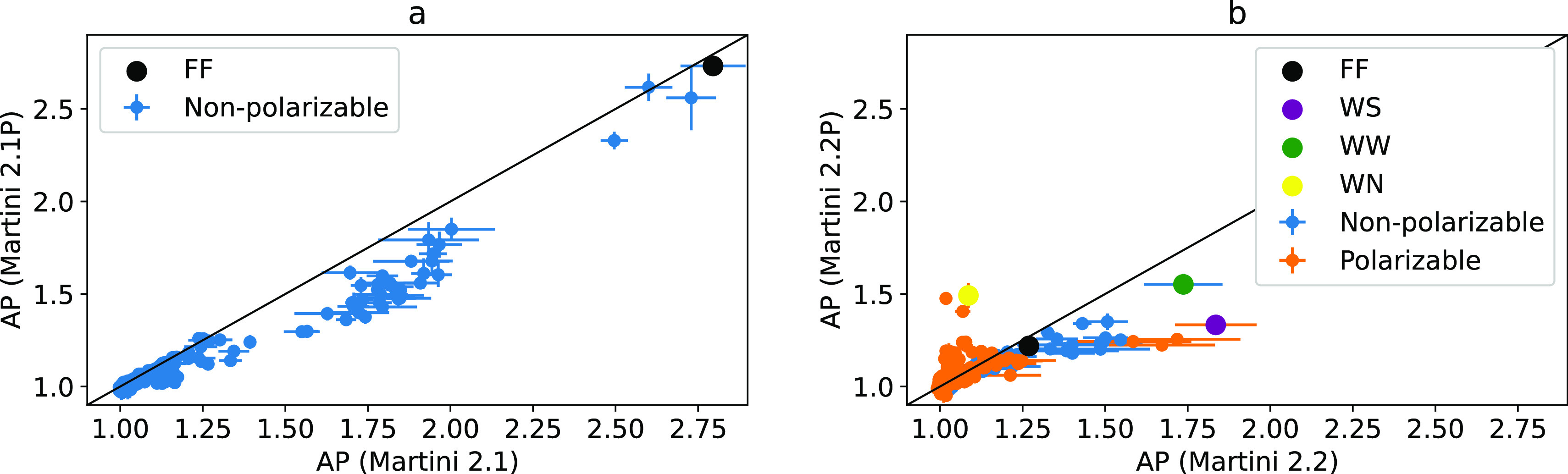
Peptides with the highest AP from each force field and FF are highlighted. (a) For Martini 2.1 AP vs 2.1P, PW decreases the AP score of midrange aggregators but does not effect the AP of the highest of lowest scoring ranges. (b) Martini 2.2 AP scores vs 2.2P, in which peptides with polarizable dummy beads are highlighted in orange and show the greatest difference in AP.

In comparing all of the Martini 2.1 and 2.1P, results we observe a slight change in the morphology of the top aggregator (FF in both cases) as well as a general trend in the decreased AP score (mean −0.05, max −0.4), which is particularly noticeable around the middle range of AP values (Figure 7a). Strong aggregators and nonaggregators are hardly affected, but where there are only weak interactions between peptides, the additional charge–charge interactions between the BB beads and PW reduce aggregation (Figure 7a).

In Martini 2.2P, embedded dipoles are introduced to improve polar-type bead representations. The effect of PW and polarizable residues between Martini 2.2 and 2.2P is considerable. Figure 7b shows that peptides with polarizable groups containing these dipoles (shown in orange) are the most divergent from the identity line, suggesting a large effect on simulating self-assembly. This case is shown starkly between dipeptides WS and WN, as they differ in only one SC bead. The dummy beads range from +0.40/–0.40 to +0.46/–0.46, and the BB–SC bond ranges from 0.25 nm (7500 kJ mol^–1^ nm^–2^) to 0.32 nm (5000 kJ mol^–1^ nm^–2^) yet they diverge in opposite directions, with AP dropping the most for WS and conversely increasing for WN.

### 3.4 Martini 3 SW/TW

Looking first to the effect of changing the solvent model from Martini 3 normal water to SW, we find that the aggregation behavior changes dramatically. The FF dipeptide no longer dissipates but instead forms a nanodisc, which is a less complex self-assembled structure (Figure 3g) with a slightly lower AP score (−0.2) than in Martini 2.1. In fact, FF, FW, WF, and WW form similar nanodiscs when using the 3SW model with a comparable solvent-accessible surface area (SASA), but since W has the largest individual SASA, there is an inflated AP score.

**Figure 8:**
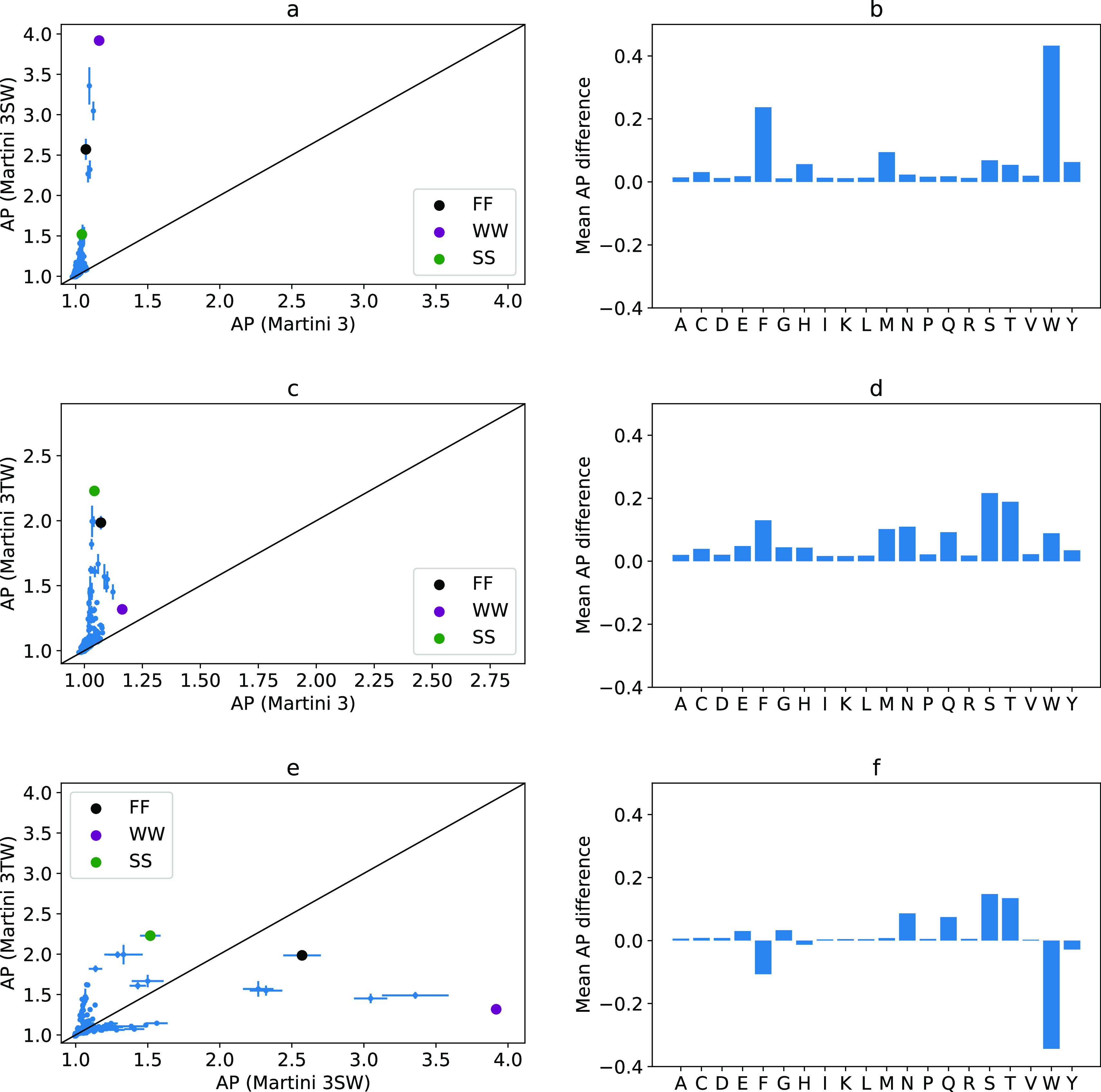
(a) Comparison between the AP score of peptide aggregation in Martini 3 and 3SW. (b) Difference in AP score contribution per amino acids. (c) Comparison between AP scores of peptide aggregation in Martini 3 and 3TW. (d) Difference in AP score contribution per amino acid. (e) Comparison between AP score of peptide aggregation in Martini 3SW and 3TW. (f) Difference in AP score contribution per amino acid.

Surprisingly, we find that in the Martini 3 TW model the best aggregator is SS (Figure 8), which is a weak aggregator in the SW model and does not aggregate in other Martini force fields. This dipeptide forms an amorphous spherical aggregate in TW, driven by a high interpeptide HB%, which is the highest of any system studied at 83%.

### 3.5 Reproducibility and Robustness of the AP Score

**Figure 9:**
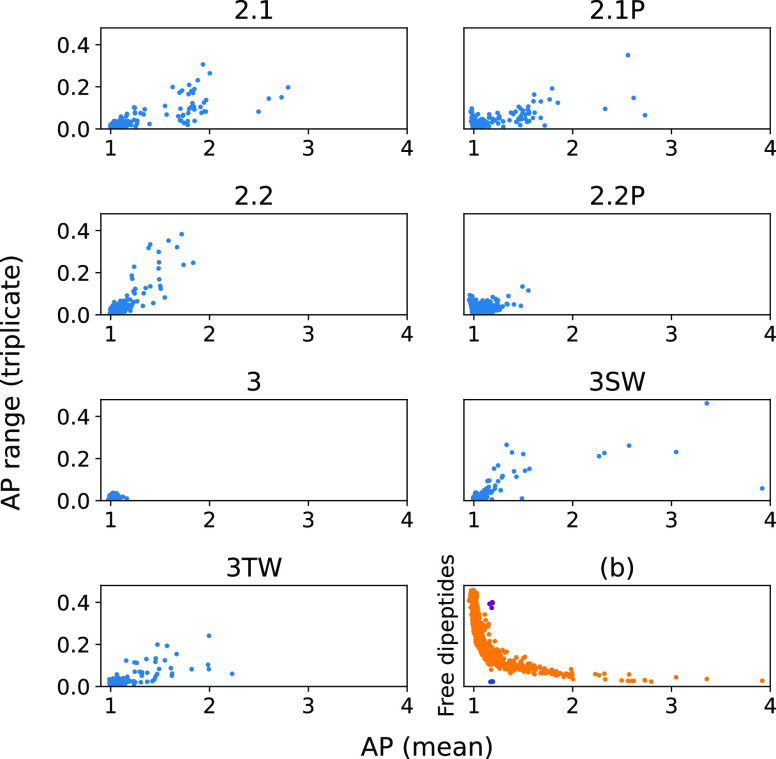
Range vs mean triplicate AP score for each force field. The uncertainty reaches a crescendo around the midpoint in the AP range for a given force field if the force field is capable of producing strong assemblers; otherwise, it increase linearly. (b) AP vs free dipeptides with KE/KD/RE/RD shown in magenta where the SC–SC self-interaction is very strong and decreases the monomer SASA without causing aggregation. EK/DK/ER/DR is shown in blue where the SC–SC interpeptide interaction is very strong, but the charge density prevents overall aggregation.

To analyze the reproducibility of the AP score as a measurement of aggregation, we compare the range of results between the triplicate simulations (Figure 9). In all cases, we observe that those dipeptides with a lower AP have a smaller range (range for AP ≤ 1.5 is 0.02). However, above this value the number of free dipeptides in solution increases only marginally (Figure 9b). This suggests that at higher AP further increases are due to specific arrangements rather than an increased number of peptides incorporated. This is due to the combined factors of the nanostructure becoming kinetically trapped and the relatively short time scales of our simulations. Where the force field does produce strong aggregators (2.1, 2.1P, 3SW, and 3TW), the best aggregator will have a low range (∼0.1) and the midrange aggregators contain systems with much larger ranges of up to 0.46. In the case of Martini 2.2 where no strong aggregates form, the range increases linearly for those systems with AP ≥ 1.6.

As demonstrated by Scott et al.,^36^ increased simulation time decreases the range in AP measurements for a given system. However, this is often not practical as AP is typically used in screening processes where single simulations of a large number of systems are desired. Nonetheless, the AP score proves to be a useful metric for screening, and the results for the low- and high-scoring peptides are reliably reproduced, which account for most cases and have proved to be a reliable target for accurate machine learning training.^3^ It is particularly useful at quickly and reliably discarding nonassemblers and providing a filtering process to focus longer simulations and experimentation on those with the greatest probability for self-assembly.

## 4 Conclusions and Outlook

In this Account, we summarize the changes that have occurred with the development of the Martini force field and how this has affected simulations of dipeptide self-assembly in water. We recognize that while extensive efforts in mitigating against problems with the earlier versions, notably overly sticky proteins, have been successful, the proficiency in simulating the self-assembly of dipeptides has been lost. Currently, the Martini 2.1 force field performs best for modeling short peptide self-assembly in aqueous environments. However, possible future iterations may reintroduce this capability through length-dependent peptide parameters or different water models. Overall, this provides insight into the interdependence of scale and aggregation and a reference for future researchers to inform their choice of force field when investigating peptide self-assembly in aqueous environments.

## Data Availability

All data underpinning this publication are openly available from the University of Strathclyde KnowledgeBase at 10.15129/dd42dfa6-8621-4c0b-a3c2-2d251c580cdf.

## Supporting Information Available

Computational procedures (PDF)
