# Supplementary material for: Short Peptide Self-Assembly in the Martini Coarse-Grain Force Field Family
Source: Acc Chem Res. 2023 Mar 3;56(6):644–54. doi: 10.1021/acs.accounts.2c00810 (PMC10035038; doi:10.1021/acs.accounts.2c00810)
Supplement: Supplementary file 1 — ar2c00810_si_001.pdf [file ar2c00810_si_001.pdf]

Supporting Information: Short Peptide Self-Assembly in the Martini Coarse Grain Forcefield Family

Alexander van Teijlingen, Melissa C. Smith, and Tell Tuttle*

Pure & Applied Chemistry, University of Strathclyde, 295 Cathedral Street, Glasgow, G1 1XL, UK

E-mail: tell.tuttle@strath.ac.uk

Phone: +44 141 548 2290

1 Bead interactions

Non-bonded interactions were calculated according to (Equation 1) where V_{LJ} is the interaction energy for a pair of atoms i,j at distance $\mathbf{r}_{ij}$ and ϵ_{ij} is the minima of potential energy at r_{min} where r_{min} is $2^{1/6}\sigma_{ij}$. With the LJ potential shifted ($r_1 = 0$) to smooth the cutoff (r_c) according to Equation 2, where $S(r_{ij})$ is the shifted potential between atoms i,j and $y(r_{ij})$ is the shifting function that tends the interaction energy towards zero.

$$V_{LJ} = 4\epsilon_{ij}\left(\left(\frac{\sigma_{ij}}{r_{ij}}\right)^{12} - \left(\frac{\sigma_{ij}}{r_{ij}}\right)^{6}\right) \times S(r_{ij}) \tag{1}$$

$$S(r_{ij}) = \begin{cases} 1 & r_{ij} < r_1 \\ 1 + y(r_{ij})^2(2y(r_{ij}) - 3) & r_1 < r_{ij} < r_c \\ 0 & r_{ij} \geq r_c \end{cases} \tag{2}$$

Where:

$$y(r_{ij}) = \frac{(r_{ij}^2 - r_1^2)}{r_c^2 - r_1^2} \tag{3}$$

Coulombic interactions (V_{crf}) are evaluated according to the reaction field algorithm (Equation 4) between the charges of atoms q_i and q_j which shifts the electronic interaction energy smoothly toward zero at the cutoff based on the local dielectric constant (ε_r), the dielectric constant beyond the cutoff (ε_{rf}) and the electric conversion factor (f) which relates electrical and mechanical properties.

$$V_{crf} \;=\; f\frac{q_i q_j}{\varepsilon_r r_{ij}}\left[1 + \frac{\varepsilon_{rf} - \varepsilon_r}{2\varepsilon_{rf} + \varepsilon_r}\frac{{r_{ij}}^3}{r_c^3}\right] - f\frac{q_i q_j}{\varepsilon_r r_c}\frac{3\varepsilon_{rf}}{2\varepsilon_{rf} + \varepsilon_r} \tag{4}$$

where:

$$f = \frac{1}{4\pi\varepsilon_0} = 138.935\,458\,\text{kJ mol}^{-1}\text{ nm } e^{-2} \tag{5}$$

Bonds, angles, dihedrals and improper dihedrals are evaluated according to Equation 6 - 9 where a force (k) is multiplied by the distance of the measure term from its equilibrium

value (b_{ij}, θ^0_{ijk}, ϕ_s, ξ_0, respectively). Dihedrals are not set for dipeptides in the Martini forcefield (except for improper dihedrals for restrained aromatic side chains), though these will still be measured to describe aggregation behavior.

$$V^b(r_{ij}) = \frac{1}{2} \times k^b_{ij}(r_{ij} - b_{ij})^2 \tag{6}$$

$$V^a(\theta_{ijk}) = \frac{1}{2} \times k^\theta_{ijk}(\theta_{ijk} - \theta^0_{ijk})^2 \tag{7}$$

$$V_d(\phi_{ijkl}) = k_\phi(1 + \cos(n\phi - \phi_s)) \tag{8}$$

$$V_{id}(\xi_{ijkl}) = \frac{1}{2}k_\xi(\xi_{ijkl} - \xi_0)^2 \tag{9}$$

2 Martini speed-up approaches

Using the Martini Straight approach, one can just use plain cutoffs, with potential modifiers that shift the entire potential up by the difference between the potential at the cutoff and zero thus eliminating the discontinuity, for both the LJ and Coulombic terms. This is more computationally efficient and able to reproduce biologically relevant phenomenon such as area per lipid (APL) of phospholipid bilayers.[1]

An explicit water model is not always necessary when using Martini. The Dry Martini version (based on 2.1) can simulate phospholipids in an implicit water system whereby the beads are reparameterized to reproduce explicit solvent systems phenomena such as APL and lateral lipid diffusion without spending up to 90% of the wall time simulating water bead interactions.[2]

3 Simulation setup

Standard Martini parameters were used throughout with the leap-frog integrator using a timestep of 25 fs and neighbor search update every 20 steps. All simulations and minimisation were performed within the GROMACS 2020.7 package.[3,4] Interactions were evaluated using a potential-shift cutoff of 1.1 nm, reaction-field electrostatics with a ε_r of 15 (2.5 was used for simulations that used the polarizable models) and a ε_{rf} of 0. The temperature was maintained at 303 K using the v-rescale thermostat separately coupled to the peptides and the rest of the system which was updated every 1 ps and pressure coupled using an isotropic Berendsen[5] barostat at 1 atm which was updated every 4 ps. Constraints were applied *via* the LINCS[6] algorithm and minimization used the steepest decent algorithm with a tolerance of 10 $kJmol^{-1}nm^{-1}$). These parameters, as they are named in GROMACS, have been summized in Table S1. 300 peptides where inserted into a 12.5 nm^3 cubic box with at least 0.3 nm spacing between each peptide and solvated with the relevant pre-equilibrated water for the forcefield. All peptide simulations were in their zwitterionic state, ions were added to neutralize net charge resulting from side chains. After each 200 ns equilibration, peptides are clustered and centered using the GROMACS trjconv utility.[3,4] Due to the relationship between the diffusion coefficients of the Martini coarse-grained and atomistic simulations, the effective simulation time is four times greater than the formal simulation time. Herein we refer to the effective simulation time.[7]

Table S1: GROMACS input parameters used to calculate AP from CG simulations in this study. The formal simulation time is 50 ns, which corresponds to 200 ns of effective simulation time in the Martini forcefield.

integrator	md	tcoupl	v-rescale
dt	0.025	nsttcouple	nstlist
nsteps	2000000	tc-grps	protein non-protein
cutoff-scheme	Verlet	tau_t	1.0 1.0
nstlist	20	ref_t	303 303
pbc	xyz	Pcoupl	Berendsen
verlet-buffer-tolerance	0.005	Pcoupltype	isotropic
rlist	1.1	nstpcouple	nstlist
coulombtype	reaction-field	tau_p	4.0
coulomb-modifier	Potential-shift-Verlet	compressibility	3e-4
rcoulomb-switch	0	ref_p	1.0
rcoulomb	1.1	gen_vel	yes
epsilon_r	15 (2.5 for 2.1P/2.2P)	gen_temp	303
epsilon_rf	0	constraints	none
vdw_type	cut-off	constraint_algorithm	Lincs
vdw-modifier	Potential-shift-verlet	lincs_order	4
rvdw_switch	0.0	lincs-iter	1
rvdw	1.1	lincs_warnangle	30

4 Descriptors

The aggregation propensity (AP, Equation 10) score was introduced in 2011[8] and is often used as a measurement to give insight into the degree of aggregation of a system by comparing the solvent accessible surface area (SASA) of the monomers at the beginning ($SASA_0$) and end of the simulation. It is used as an indicator of the prerequisite aggregation to self-assembly to determine numerically which dipeptides are aggregating under each forcefield. As an addition to forcefield discrimination, we evaluate the robustness of the AP score as a metric in terms effectiveness and reliability in measuring aggregation across repeat studies.

$$AP = \frac{SASA_0}{SASA} \tag{10}$$

Along with the AP score we evaluate the usefulness of Martini simulations *via* two other descriptors, radius of gyration (R_g, Equation 11) and hydrogen bonding percentage (HB%,

Equation 12). The former has a well documented history as means of measuring the compactness of proteins from the mean distances of particles (r_k) from their center of mass (r_{mean}) and has been used to measure sphericalness of molecular aggregates[9] and elongation of aggregate structures.[10]

$$R_g = \sqrt{\frac{1}{N}\sum_{k=1}^{N}(r_k - r_{mean})^2} \tag{11}$$

To aid in determining the degree aggregation as driven by hydrophobic effects *vs* hydrogen bonding we measure HB%, a metric derived from that reported by van Lommel *et al.*[11] In this study HB% has been defined as the percentage of donor and acceptor beads involved in hydrogen bonding with corresponding beads in other dipeptides. The cutoff distance was set to 4.7 Å which reflects the coarse-grained nature of the systems.

$$HB\% = \frac{\sum_i \sum_{j \neq i} \begin{cases} 1 & , r_{ij} \leq 4.7\,\text{Å} \\ 0 & , r_{ij} > 4.7\,\text{Å} \end{cases}}{N} \times 100\% \tag{12}$$

5 File Archive

All data underpinning this publication are openly available from the University of Strathclyde KnowledgeBase at https://doi.org/10.15129/dd42dfa6-8621-4c0b-a3c2-2d251c580cdf

References

1 De Jong, D. H.; Baoukina, S.; Ingólfsson, H. I.; Marrink, S. J. Martini straight: Boosting performance using a shorter cutoff and GPUs. *Comput. Phys. Commun.* **2016**, *199*, 1–7.

2 Arnarez, C.; Uusitalo, J. J.; Masman, M. F.; Ingólfsson, H. I.; De Jong, D. H.; Melo, M. N.; Periole, X.; De Vries, A. H.; Marrink, S. J. Dry martini, a coarse-grained force field for lipid membrane simulations with implicit solvent. *J. Chem. Theory Comput.* **2015**, *11*, 260–275.

3 Van Der Spoel, D.; Lindahl, E.; Hess, B.; Groenhof, G.; Mark, A. E.; Berendsen, H. J. GROMACS: Fast, flexible, and free. *J. Comput. Chem.* **2005**, *26*, 1701–1718.

4 Abraham, M. J.; Murtola, T.; Schulz, R.; Páll, S.; Smith, J. C.; Hess, B.; Lindahl, E. GROMACS: High performance molecular simulations through multi-level parallelism from laptops to supercomputers. *SoftwareX* **2015**, *1-2*, 19–25.

5 Berendsen, H. J.; Postma, J. P.; Van Gunsteren, W. F.; Dinola, A.; Haak, J. R. Molecular dynamics with coupling to an external bath. *J. Chem. Phys.* **1984**, *81*, 3684–3690.

6 Hess, B. P-LINCS: A parallel linear constraint solver for molecular simulation. *J. Chem. Theory Comput.* **2008**, *4*, 116–122.

7 Marrink, S. J.; Risselada, H. J.; Yefimov, S.; Tieleman, D. P.; De Vries, A. H. The MARTINI force field: Coarse grained model for biomolecular simulations. *J. Phys. Chem. B* **2007**, *111*, 7812–7824.

8 Frederix, P. W. J. M.; Ulijn, R. V.; Hunt, N. T.; Tuttle, T. Virtual Screening for Dipeptide Aggregation: Toward Predictive Tools for Peptide Self-Assembly. *J. Phys. Chem. Lett.* **2011**, *2*, 2380–2384.

9 Tang, Y.; Bera, S.; Yao, Y.; Zeng, J.; Lao, Z.; Dong, X.; Gazit, E.; Wei, G. Prediction and

characterization of liquid-liquid phase separation of minimalistic peptides. *Cell Reports Phys. Sci.* **2021**, *2*, 100579.

10 Marrink, S. J.; Tieleman, D. P. Perspective on the Martini model. *Chem. Soc. Rev.* **2013**, *42*, 6801.

11 Van Lommel, R.; Zhao, J.; De Borggraeve, W. M.; De Proft, F.; Alonso, M. Molecular dynamics based descriptors for predicting supramolecular gelation. *Chem. Sci.* **2020**, *11*, 4226–4238.
